# The kynurenine pathway is involved in bacterial meningitis

**DOI:** 10.1186/s12974-014-0169-4

**Published:** 2014-10-02

**Authors:** Leonam G Coutinho, Stephan Christen, Caroline L Bellac, Fabrícia Lima Fontes, Fladjule Rejane Soares de Souza, Denis Grandgirard, Stephen L Leib, Lucymara F Agnez-Lima

**Affiliations:** Departamento de Biologia Celular e Genética, Universidade Federal do Rio Grande do Norte, UFRN, Campus Universitário, Lagoa Nova, Natal, RN Brazil; Institute for Infectious Diseases, University of Bern, Friedbühlstrasse 51, PO Box 61, CH-3010 Bern, Switzerland

**Keywords:** Bacterial meningitis, Kynurenine, IDO activity, CSF, Cytokines, AADAT + 401C/T

## Abstract

**Background:**

Bacterial meningitis (BM) is characterized by an intense host inflammatory reaction, which contributes to the development of brain damage and neuronal sequelae. Activation of the kynurenine (KYN) pathway (KP) has been reported in various neurological diseases as a consequence of inflammation. Previously, the KP was shown to be activated in animal models of BM, and the association of the SNP AADAT + 401C/T (kynurenine aminotransferase II - KAT II) with the host immune response to BM has been described. The aim of this study was to investigate the involvement of the KP during BM in humans by assessing the concentrations of KYN metabolites in the cerebrospinal fluid (CSF) of BM patients and their relationship with the inflammatory response compared to aseptic meningitis (AM) and non-meningitis (NM) groups.

**Methods:**

The concentrations of tryptophan (TRP), KYN, kynurenic acid (KYNA) and anthranilic acid (AA) were assessed by HPLC from CSF samples of patients hospitalized in the Giselda Trigueiro Hospital in Natal (Rio Grande do Norte, Brazil). The KYN/TRP ratio was used as an index of indoleamine 2,3-dioxygenase (IDO) activity, and cytokines were measured using a multiplex cytokine assay. The KYNA level was also analyzed in relation to AADAT + 401C/T genotypes.

**Results:**

In CSF from patients with BM, elevated levels of KYN, KYNA, AA, IDO activity and cytokines were observed. The cytokines INF-γ and IL-1Ra showed a positive correlation with IDO activity, and TNF-α and IL-10 were positively correlated with KYN and KYNA, respectively. Furthermore, the highest levels of KYNA were associated with the AADAT + 401 C/T variant allele.

**Conclusion:**

This study suggests a downward modulatory effect of the KP on CSF inflammation during BM.

## Introduction

Despite the advances in antimicrobial and intensive care therapies against bacterial meningitis (BM), high mortality and morbidity rates have been observed [1]. Neurological sequelae, such as motor abnormalities, seizures, learning and memory impairment and mental retardation, are frequently reported after the disease. Cellular damage, mainly in the cerebral cortex, hippocampus and inner ear are the histomorphological correlates of these neurofunctional deficits [2-4].

Bacterial invasion and proliferation within the cerebrospinal fluid (CSF) induce an intense inflammatory response that leads to the activation of several metabolic pathways. One such pathway is the kynurenine (KYN) pathway (KP), which has been shown to be activated in experimental pneumococcal meningitis [5,6]. The KP is the major route for tryptophan (TRP) oxidative degradation, and this pathway is involved in several diseases of the nervous system, including cancer, inflammatory disorders and neurodegenerative diseases [7-10]. The first step in the KP is the degradation of TRP catalyzed by indoleamine 2,3-dioxygenase (IDO) to generate formylkynurenine, which is rapidly converted into KYN by the action of kynurenine formamidase. KYN can be partially metabolized to kynurenic acid (KYNA) and anthranilic acid (AA) by kynurenine aminotransferase and kynureninase, respectively, and in part converted to 3-hydroxykynurenine (3-HK) by kynurenine 3-monooxygenase. Kynureninase also converts 3-HK to 3-hydroxyanthranilic acid (3-HAA), the substrate for 3-hydroxyanthranilic acid oxidase, which generates quinolinic acid (QUIN), the major substrate for NAD^+^*de novo* synthesis [8,9].

In the inflamed brain, TRP is metabolized through the KP to form neuroactive metabolites such as QUIN, an agonist of excitotoxic NMDA receptors, showing a cytotoxic effect, and KYNA, a neuroprotector antagonist of these NMDA receptors [10,11]. Because the KP enzymes are differentially expressed in several cell types, quantitative differences in the production of neurotoxic and neuroprotective metabolites are observed [12-15], and an unbalance in the KP has been associated with several CNS diseases [7-10]. In an animal model of BM, the chemical inhibition of the KP led to decreased cellular NAD levels and increased apoptosis in the hippocampus [6]. This was associated with high activity of PARP-1, a DNA repair protein activated by DNA strand breaks caused by oxidative stress during BM. PARP-1 uses NAD^+^ as a cofactor, and its high activity induces energetic depletion, leading to the cell death and neuronal injury typical of BM [16]. Due to the involvement in NAD^+^*de novo* synthesis and KYNA production, the KP is possibly a neuroprotective pathway in BM, despite its neurotoxic metabolites [6].

Important immunomodulatory properties, mainly related to the immunosuppressive effect of IDO, have also been attributed to this pathway [8,9,17]. A feedback mechanism in modulating the immune responses has been proposed because proinflammatory stimuli activate the KP and an anti-inflammatory effect mediated by KYNA has been observed [8,18,19]. IDO is preferentially induced by interferons, with IFN-γ being the main cytokine involved in its induction. A synergistic effect of IL-1β, TNF-α and IL-6 in IDO induction was also described. However, there is evidence that IDO expression can also be induced by an IFN-γ-independent mechanism that involves NF-κB and stress-activated mitogen-activated protein (MAP) kinases, such as p38 and c-Jun N-terminal kinase (JNK). Conversely, an immune-suppressive effect of the KP has also been described. QUIN and 3-HAA induce the selective apoptosis of TH1 cells through the activation of the caspase pathway; B and NK cells are also susceptible to these compounds. In addition, the expression of IDO in dendritic cells induces the generation of regulatory T-cells. Thus, high levels of IDO will result in a decline of TH1 response, accompanied by an enhanced TH2 response. Moreover, KYNA shows an inhibitory effect on TNF-α at the transcriptional level and as a ligand of GPR35. This inhibition of TNF-α by KYNA may be an important factor in its neuroprotection [8,9].

In a previous work, we identified the association of the SNP AADAT + 401C/T (kynurenine aminotransferase II -– KAT II) with the host immune response to BM, and our results suggested that this SNP may affect the host’s ability to recruit leukocytes to the infection site [20]. This evidence raises the hypothesis that the KP plays an important role in the pathogenesis of BM in humans. In the present work, we measured the concentrations of metabolites of the KP in CSF samples from patients with meningitis and analyzed their correlation with cytokines, inflammatory modulators previously reported to regulate the pathogenesis of BM. We also investigated the correlation of KAT II genotypes with KYNA levels and the disease.

## Material and methods

### Case selection and sample collection

This is a prospective study involving 28 patients with the clinical suspicion of meningitis who were admitted to the Giselda Trigueiro Hospital in Natal (Rio Grande do Norte, Brazil), a reference center for infectious diseases. Ethical approval for this study was given by Committees on Medical Ethics of the Giselda Trigueiro Hospital and by the National Committee in Ethics (CONEP) with number 0052.1.051.000-05. Informed consent was obtained from each patient participating in this study. For child patients, informed consent was obtained from their parents or legal guardians.

Lumbar puncture (LP) was performed to obtain CSF for diagnostic purposes. Twenty-eight CSF samples were collected at the time of admission. Immediately after sampling, the CSF was kept at 4°C before centrifugation (400 × g, 5 minutes, 4°C). The supernatant were immediately frozen and stored at −80°C until assayed. The CSF samples were anonymized.

Thirteen patients were diagnosed with BM by the following criteria: (1) positive CSF bacterial culture, (2) detection of the pathogen in the CSF by gram staining plus clinical signs (acute onset, fever, meningeal irritation) and/or (3) positive blood culture or Gram stain in the presence of clinical signs of meningitis, (4) positive bacterial antigen detection in the CSF or blood using the latex agglutination test, with clinical signs of meningitis, and (5) clinical signs plus CSF parameters of increased protein content (>40 mg/dL), reduced glucose levels (<40 mg/dL) and the presence of CSF pleocytosis (≥500 cells/mm^3^), with predominantly polymorphonuclear granulocytes (PMN). Aseptic meningitis (AM) was diagnosed in seven patients by acute onset, fever, meningeal irritation signs, mild increase in protein content and normal glucose levels and the absence of the detection of bacterial pathogens. Because LP is a very invasive procedure, CSF samples were obtained only from patients undergoing procedures for the diagnosis of meningitis. Eight patients had a negative diagnosis for CNS infection and were included as non-meningitis controls (NM) (Table 1). Patients with confirmed acquired immunodeficiency syndrome were excluded from this study.

**Table 1:** **Cerebrospinal fluid (CSF) parameters of patients with bacterial meningitis (BM) and aseptic meningitis (AM)**

**Groups**	**Non-meningitis, n = 8**	**Bacterial meningitis, n = 13**	**Aseptic meningitis, n = 7**
**Cell count** ^e^	2.4 ± 0.8	2,468 ± 794.5^ab^	81.3 ± 38.0
**Protein** ^e^	23.5 ± 8.1	171.2 ± 59.2^ab^	52.7 ± 20.8
**Glucose** ^f^	72.8 + 8.3^d^	29.4 ± 5.7	53.2 ± 5.5
**Age (months)** ^g^	440.6 ± 85.8^c^	159.4 ± 39.8	280.9 ± 72.5

Mean ± SEM.

^a^ *P < 0*.001 and ^b^ *P < 0*.05 significant values in the comparison to non-meningitis (NM) and AM respectively, and ^c^ *P < 0*.01 ^d^ *P < 0*.001 in the comparison of BM. ^e^ *P < 0*.001 to one-way analysis with Kruskal-Wallis correction. ^f^ *P < 0*.001 and ^g^ *P < 0*.05 to one-way analysis of variance (ANOVA).

### Analysis of kynurenine by HPLC

CSF was analyzed by high-performance liquid chromatography (HPLC) to quantify the levels of TRP, KYN, KYNA and AA. Briefly, CSF samples were mixed 4:1 with 6% perchloric acid (PCA) to precipitate proteins. The acidified samples were centrifuged 10,000 × g at 4°C for 10 minutes, and the supernatant was filtered through 0.2-μm nylon membranes. An 80-μL aliquot of the filtrate was applied onto a C18 reverse-phase HPLC column (Supelcosil LC-18-DB, 15 cm × 4.6 mm, 3 μm; Supelco, Buchs, Switzerland) with a guard column (Supelguard LC-18-DB, 2 cm; Supelco, Buchs, Switzerland). TRP metabolites were eluted isocratically at a flow rate of 0.8 mL/minute with a mobile phase consisting of 100 mM zinc acetate and 3% acetonitrile (v/v), pH 6.2 [17,18]. TRP and KYN were detected by UV absorption at 280 nm and 360 nm, respectively (L-4250 UV VIS Detector, Merck Hitachi). AA and KYNA were detected by fluorescence (F-1080 Fluorescence Detector, Merck Hitachi) at an excitation of 344 nm and emission of 400 nm. Chromatograms were generated and analyzed using D-7000 HPLC System Manager software.

### Cytokine multiplex measurements

The concentration of six cytokines (TNF-α, IL-6, IL-1β, IFN-γ, IL-10 and IL-1Ra) was assessed in CSF samples using the human cytokine Linco*plex* Kit (HCYTO-60 K, Lincoplex®, Linco Research Inc., St Charles, MA, USA) with Luminex Technology (Bio-Plex 200 suspension array system, Bio-Rad, Hercules, CA, USA). The assay was performed according to the manufacturer’s instructions. Samples were diluted to fit within the dynamic range of the assay. The cytokine concentrations were calculated by the Bio-Plex Manager software using a five-parametric logistic standard curve derived from the recombinant cytokine standards provided in the kit.

### Statistical analysis

Data were analyzed by analysis of variance (ANOVA), with the Kruskal-Wallis test if needed (Prism 4.0, GraphPad, San Diego, CA, USA). A *P* < 0.05 was considered to be statistically significant. Multiple comparisons with Tukey’s test were performed if the distribution was normal. Differences between two groups were analyzed with the Mann–Whitney test when the distribution was not normal and with Dunn’s post test when more than two groups were analyzed. The table and graph data are presented as the median ± interquartiles when not following a Gaussian distribution and the mean ± SEM when following a Gaussian distribution.

## Results

### Patients

Patients were divided into three groups, BM, AM and NM, according to the diagnostic criteria (Table 1). The CSF parameters found in the BM group were significantly different in comparison to those in the AM and NM groups. BM was diagnosed in 13 patients, and the causative pathogens were *Streptococcus pneumoniae* (n = 7), *Neisseria meningitidis* (n = 1) and *Proteus mirabilis* (n = 1); for *4* patients, the bacterial species could not be identified. Three patients died during hospitalization. Two belonged to the BM group and 1 to the AM group.

### Concentration of tryptophan and KYN metabolites

**Figure 1:**
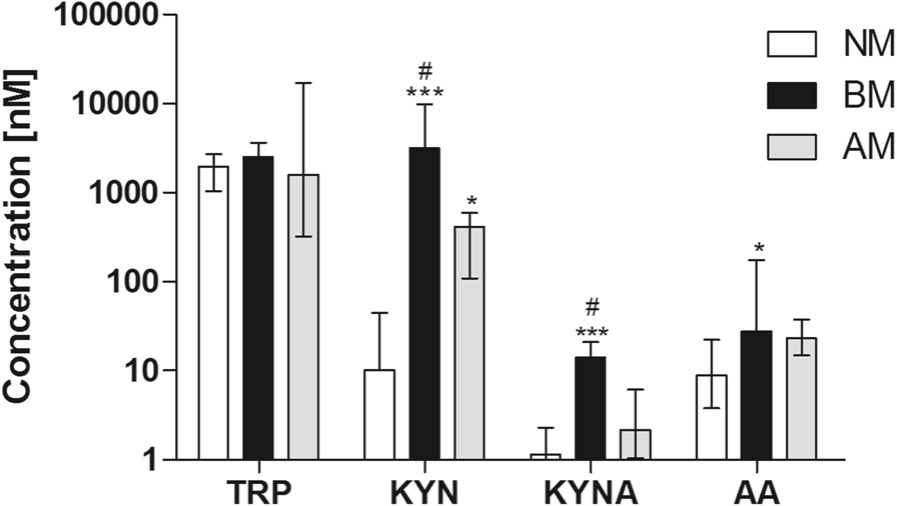
**Tryptophan (TRP), kynurenine (KYN), kynurenic acid (KYNA) and anthranilic acid (AA) concentrations in cerebrospinal fluid (CSF) samples from human patients.** Each metabolite was analyzed independently between groups. Differences between groups were analyzed with multiple comparison using Dunn’s post test. *P*-value to one-way analysis with Kruskal-Wallis correction was lower than *P < 0*.05 to all metabolites, except TRP. Values are expressing as median ± interquartiles. ***P < 0.001 and *P < 0.05 compared to non-meningitis (NM); ^#^ *P <* 0.05 compared to aseptic meningitis (AM). Values that were below the detection limit were adjusted to the limit.

Compared to the levels found in NM, a significant increase of KYN was observed in BM (*P* < 0.001) and AM (*P* < 0.05) (Figure 1). The KYNA levels in CSF were found to be significantly increased in BM compared to NM and AM (*P* < 0.001 and *P* < 0.01, respectively) (Figure 1). The CSF concentrations of AA showed significantly increased levels in BM versus NM (Figure 1).

**Figure 2:**
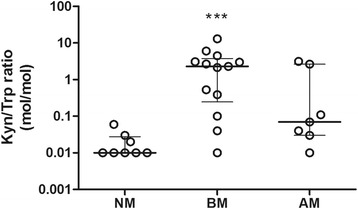
**Kynurenine to tryptophan (KYN/TRP) ratio indicates indoleamine 2,3-dioxygenase (IDO) activity.** Increased enzymatic activity was found for IDO in cerebrospinal fluid (CSF) samples. Values are expressing as median ± interquartiles. Data showed *P <* 0.01 to one-way analysis with Kruskal-Wallis correction. Differences between two groups were analyzed with Dunn’s post test. Values that were below the detection limit were adjusted to the limit.

The ratio of KYN/TRP was calculated as an index of IDO activity in the CSF samples. The ratio between KYN and TRP determined in the CSF samples was significantly increased in BM compared to NM (*P* < 0.001) (Figure 2). AM showed no significant difference with regard to the KYN/TRP ratio compared to NM. KYN concentrations of NM patients that were below of the detection limit (10 nM) were adjusted to this value.

### Concentrations of cytokines

**Figure 3:**
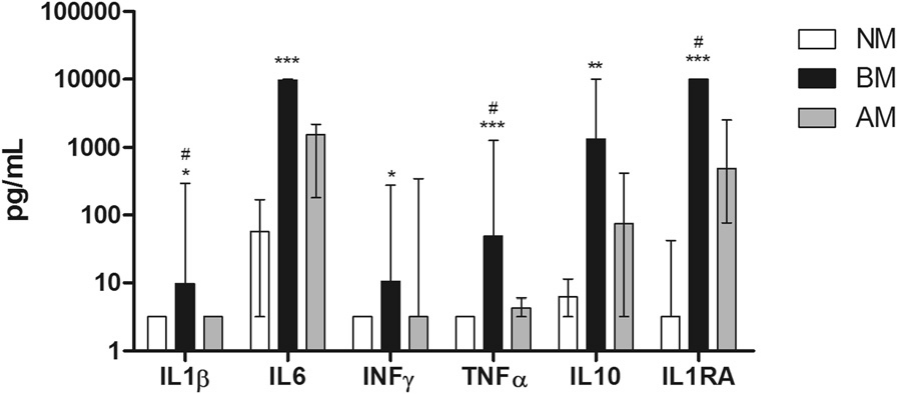
**Comparison of the cytokines levels in the cerebrospinal fluid (CSF) during meningitis.** Values are expressing as median ± interquartiles. Data showed *P <* 0.01 to one-way analysis with Kruskal-Wallis correction. Differences between two groups were analyzed with Dunn’s post test. ***P < 0.001, **P < 0.01 and *P < 0.05 compared to non-meningitis (NM). ^#^ *P <* 0.05 compared to aseptic meningitis (AM).

The concentrations of the six cytokines, IL-1β, IL-6, TNF-α, IFN-γ, IL-10 and IL-1Ra, were significantly upregulated in CSF during BM when compared to the NM control group (Figure 3). Despite the AM group show an increase in some cytokines, for example, IL-6, TNF-α, IL-10 and IL-1Ra, significant differences compared to the NM group were not observed.

Furthermore, a positive correlation between the concentrations of IFN-γ and IL-1Ra with IDO activity was found in the BM patients, as well as between TNF-α *versus* KYN and KYNA *versu*s IL-10 (Table 2).

**Table 2:** **Correlation of kynurenine to tryptophan (KYN/TRP) ratio and KYN metabolites with cerebrospinal fluid (CSF) cytokine levels in patients with bacterial meningitis (BM)**

	**IFN-γ**	**TNF-α**	**IL-10**	**IL-1RA**
**KYN/TRP ratio**	0.77	-	-	0.63
**KYN**	-	0.77	-	-
**KYNA**	-	-	0.57	-
***P-value***	*P <* 0.01	*P <* 0.01	*P <* 0.05	*P <* 0.05

-Values mean the results obtained from Spearman’s correlation. Data did not follow Gaussian distribution. *Abbreviation:* *KYNA* kynurenic acid.

### Correlation of genotype and KYNA levels

**Figure 4:**
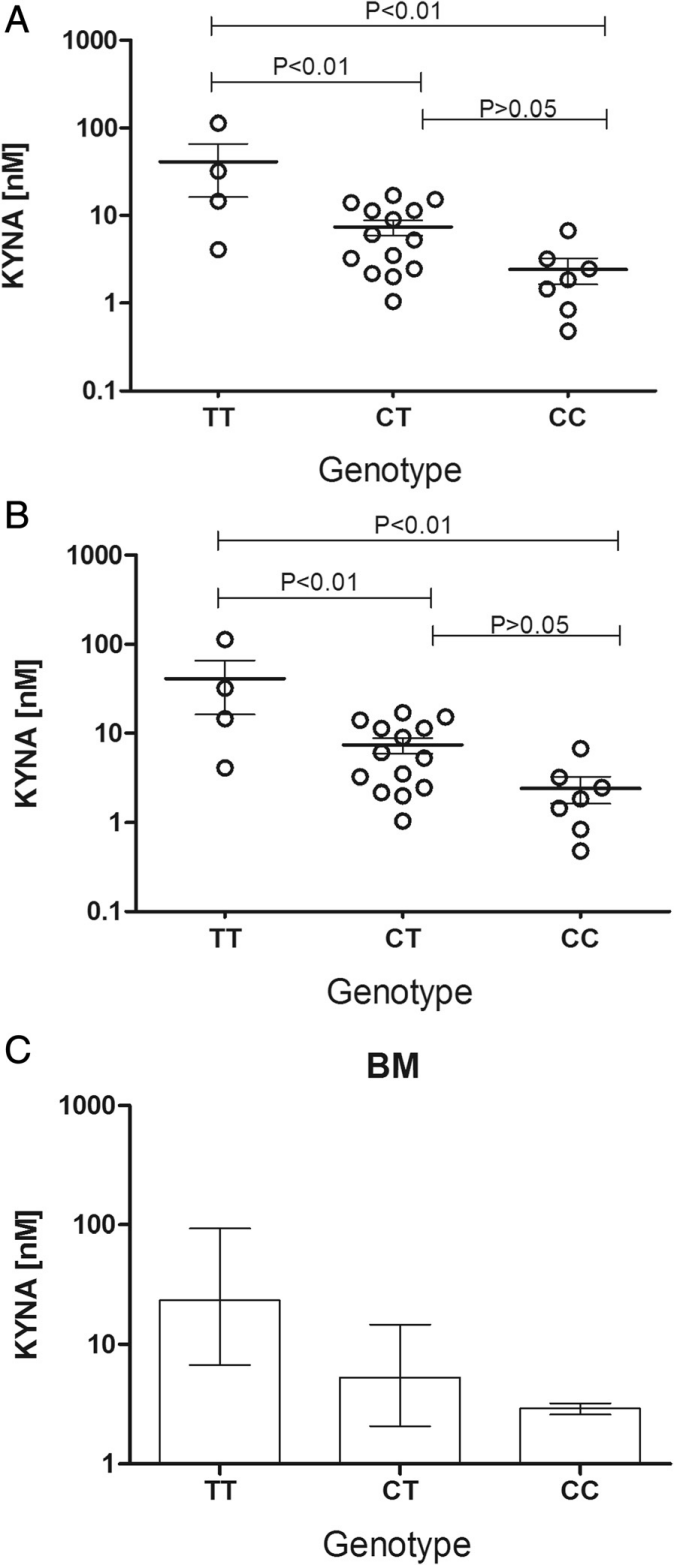
**Influence of the individual genotype on the levels of kynurenic acid (KYNA).** Patients showing polymorphisms in the *KAT II* gene demonstrated increase in the KYNA concentration. All patients in the study were split according genotype. **(A)** Patients homozygous for variant allele (TT) showed higher KYNA concentration than heterozygous (CT) and homozygous for wild type allele (CC). **(B)** Patients with genotype CT were divided in two groups according to disease showing the contribution of bacterial meningitis (BM) combined with the variant allele in the elevated concentration of KYNA. **(C)** Patients with BM were split according to their genotype and KYNA levels were compared but no significant difference was found. Values were expressed as mean ± SEM in graph **(A)** and median ± interquartiles in **(B)** and **(C)**. Data in graph **(A)** showed Gaussian distribution and were analyzed with one-way analysis of variance (ANOVA) with post test Tukey’s test to compare all genotypes, while in graphs **(B)** and **(C)**, differences between groups were analyzed with the Mann–Whitney test and Dunn’s post test, respectively.

In a previous work, we obtained the genotype of these patients in relation to SNP AADAT + 401C/T (KAT II) and observed a high frequency of the T allele and TT genotype in BM patients [20]. As the number of patients in our study was small for polymorphism association, we first analyzed all the patients in the study only according to genotype. In this first analysis, the patients carrying the T variant allele showed an increased KYNA concentration (Figure 4A). Thereafter, the largest group of individuals, in this case the heterozygotes (CT), was divided into two groups considering their disease, as shown in Figure 4B. The obtained data showed the contribution of acute BM combined with the variant allele in the increase of KYNA levels. Individuals with acute BM were also analyzed according to genotype, and the highest KYNA levels were observed to be associated with the TT genotype, however no significant difference was observed (Figure 4C).

## Discussion

CSF concentrations of KP metabolites have been demonstrated in a variety of neurological diseases [7-10]. In the present study, the concentration of TRP and three KYN metabolites, IDO activity and cytokines were assessed in the CSF of patients with meningitis. The obtained data demonstrated an increase in KYN metabolites in CSF and suggested a role of IDO, kynureninase and anthranilate 3-hydroxylase in accelerating the synthesis of KYN, KYNA and AA in human BM patients, corroborating the findings from animal models [5,6].

Inflammation in the CNS results in the recruitment of activated T-cells and macrophages from the periphery into the neural parenchyma [21]. Activated T-cells produce cytokine mediators of the inflammatory response, including the macrophage-activating cytokine IFN-γ, which induces IDO activity [22,23]. In our work, TRP levels are not altered in the CSF of patients with BM or AM compared to NM. However, efficient conversion of TRP to KYN could be documented. IDO activity in the CSF was significantly higher only in the BM group, in agreement with the low IFN-γ level observed in the AM group. Conversely, a strong immune response during BM has been positively correlated with IDO activity. In some non-inflammatory and inflammatory diseases including BM, the CSF QUIN, KYN and KYNA levels were also correlated with immune markers, such as neopterin, white blood cell counts and lgG levels, indicating a close relationship between the KP and the brain’s inflammatory response [24].

Reports linking increased KYN metabolism with neuronal injury, particularly during neuroinflammatory disease, have emerged. This neuronal injury would be mainly attributed to the production of neurotoxic metabolites and oxidative molecules, such as superoxide anion and hydrogen peroxide, during the metabolism of KYN [7-10,25,26]. In contrast, *in vitro* studies have attributed antioxidant activity to certain KP metabolites [27-29].

Due to these roles, the KP has been investigated as a target for therapeutic intervention. The pharmacological modulation of KYN metabolism can affect *de novo* NAD synthesis [6,30]. NAD is involved in many metabolic processes, being an essential co-factor for several enzymes, including PARP-1, which plays a crucial role in the development of meningitis-associated central nervous system complications [16]. In this sense, the KP intervention can affect NAD production and consequently PARP-1 activity, as the inhibition of the KP induced an increase in apoptosis in a BM animal model [6].

During the course of bacterial meningitis, anti-inflammatory cytokines are produced to mitigate the induced inflammation [4,31]. Some studies have proposed that cells of the immune system use a bidirectional communication in which TRP catabolism through IDO activity drives the generation of IL-10-producing regulatory T-cells [8,9,18,19]. In BM patients, we observed a positive correlation between IDO activity and the inflammatory cytokines IFN-γ and IL-1Ra (Table 2). Although each cytokine displays different roles in the inflammation during BM, IFN-γ seems to have a crucial role, mainly during pneumococcal meningitis [32-35], showing to be involved in deleterious proinflammatory effects [34,35]. Concomitantly, KYN metabolites showed a positive correlation with TNF-α and KYNA metabolites with IL-10. In contrast, AM did not show a strong immune response or a high level of IL-10, despite a significant amount of KYN in the comparison with the NM group. Therefore, we propose that KYN metabolites alone are not able to trigger the cascade of inflammatory mediator production. In animal models, long-term diurnal hypoactivity and nocturnal hyperactivity have been reported in wild-type mice, while these changes were not observed in IDO1(−/−) mice. However, no protection against developing long-term cognitive deficits was observed in IDO deficient mice. These data suggest that KP may be involved in some behavioural sequelae of pneumococcal meningitis and may acts synergistically, or independently of, other metabolic pathways to cause different types of neurological sequelae [36].

Recently, our group demonstrated that a polymorphism in the *kynurenine aminotransferase II* gene (AADAT + 401C/T) was associated with reduced levels of TNF-α, IL-1β, IL-6, MIP-1 /CCL3 and MIP-1/CCL4 in BM patients; a reduction in cell count with a high correlation with cytokine and chemokine levels was also observed, suggesting a reduced leukocyte recruitment ability in these patients [20]. This SNP is located in a putative exonic splicing silencer (ESS), which may result in a quantitative increase in the production of mRNAs and protein [37]. In agreement with this proposition, analyzing the data from KYNA measurements in the patients homozygous for the TT genotype and comparing with the CC homozygous, we observed an increase in KYNA levels (Figure 4). Interestingly, our results demonstrated a positive correlation between KYNA and IL-10, an anti-inflammatory cytokine that was previously reported to down-regulate the expression of proinflammatory cytokines such as TNF-α. Similar data were obtained in a study carried out by Hsieh *et al*., showing that KYNA improves the outcomes of heatstroke in rats. In this model, KYNA inhibited the expression of inflammatory molecules such as TNF-α and ICAM-1 and enhanced IL-10 levels [19]. Corroborating the findings regarding neuroprotection, the reduced production of KYNA in astrocytes has been proposed to increase neurological symptoms of cerebral malaria [38]. KYNA was identified as a ligand for the receptor for GPR35, as it was able to attenuate LPS-induced TNF-α secretion in a dose-dependent manner. Because the TRP metabolic pathway is activated by proinflammatory stimuli, the anti-inflammatory effect of KYNA suggests an interesting feedback mechanism in modulating immune responses [18].

In addition to cytokines, other molecules can regulate the KP. There are known interactions between TRP depletion and nitric oxide (NO) production. Several findings have demonstrated that NO is able to inhibit the IDO enzyme by direct interaction or accelerating proteasomal degradation [39,40]. Furthermore, as shown in the report from Samelson-Jones and Yeh, several cellular factors, including pH, redox environment, NO, and L-Trp abundance can be considered as additional aspects in the regulation of IDO activity [41].

Altogether, these findings suggest that IDO has prominent importance during bacterial meningitis due to the following: its capacity to maintain PARP-1 activity through NAD synthesis, protecting against cell death [6,16]; the generation of reactive oxygen species (ROS), helping the defense mechanisms in addition to maintaining IDO activity [8-10,26]; its capacity to produce some antioxidant metabolites [27]; and regulating the immune response via the known vessel-relaxing ability which KYN possess during inflammation and other immunosuppressive mechanisms [18,19,42]. We also suggest that AADAT + 401C/T patients can present a better outcome during BM disease via a reduced inflammatory response and increased capacity of the neuroprotective role of KYNA, which requires further investigation. Conversely, this reduced response to a pathogen at the beginning of infection may favor invasion of the microorganism, increasing the susceptibility to BM. However, the small number of patients analyzed and the heterogeneity between the groups are limitations of our work, making necessary further studies with a larger cohort for a better understanding of the role of KYNA in the outcome of bacterial meningitis.

In conclusion, our results demonstrate a specific upregulation of the KP in BM patients and suggest that KYN metabolites contribute directly to the disease. Despite the neurotoxic properties reported in the literature to date, we propose a positive and essential role of this metabolic pathway in BM.
